# Supplementary material for: Diet and Lifestyle Factors and Risk of Atherosclerotic Cardiovascular Disease—A Prospective Cohort Study
Source: Nutrients. 2021 Oct 27;13(11):3822. doi: 10.3390/nu13113822 (PMC8622601; doi:10.3390/nu13113822)
Supplement: Supplementary file 1 [file nutrients-13-03822-s001.zip › nutrients-1434463-supplementary.pdf]

Table S1. Validation of diagnosis of atherosclerotic cardiovascular disease

Validation of diagnosis of coronary artery disease	One hundred patients with diagnosis of coronary artery disease were randomly selected for the validation procedure using patient record data. Among 100 patients, 96 had acute myocardial infarction, of which 29 had ST-Elevation Myocardial Infarction (STEMI) and 67 had non-STEMI (NSTEMI). One patient died due to acute circulatory insufficiency due to unclear cause, but did not undergo autopsy. Three patients did not have acute myocardial infarction; one had transitory cerebral ischemic attack, one had acute pulmonary embolism, and one had acute biliary duct stone disease. Hence, acute myocardial infarction was confirmed in 96% of cases.
Validation of diagnosis of ischemic stroke	One hundred patients with diagnosis of ischemic were randomly selected for the validation procedure using patient record data. Among 100 patients, 89 had stroke and 87 had IS. Two patients had intra-cerebral hemorrhage. It was unclear if one patient with fatal outcome had stroke or not, and autopsy was not undertaken. Of the ten patients that did not have stroke, four had transitory ischemic attack due to intra-cerebral thrombosis. Six patients did not have a cerebral ischemic event due to epilepsy (n=1), primary progressive aphasia (n=1), syncope (n=1), disorientation (n=1), headache (n=1) and acute lower limb ischemia (n=1). Among 87 with ischemic

	stroke, the distribution of causes were the following: Intra-cerebral thrombosis (n=43; 49.4%), embolization secondary to atrial fibrillation (n=31; 35.6%), embolization due to carotid artery stenosis (n=7; 8.0%), carotid artery dissection (n=2), embolization secondary to endocarditis (n=1), unclear if symptomatic carotid artery stenosis or intra-cerebral thrombosis (n=2) and unclear if cardiac arrhythmias or intra-cerebral thrombosis (n=1). Among the 98 evaluable patients, 56 (57%) had an atherosclerotic cause of disease. The diagnosis of ischemic stroke was confirmed in 89% (87/98) of cases.
Validation of diagnosis of carotid artery disease diagnosis	One hundred patients with diagnosis of carotid artery disease were randomly selected for the validation procedure using patient record data. Among 100 patients, 57 had symptomatic ($\geq$ 60% stenosis of the internal carotid artery on color doppler ultrasound) and 42 asymptomatic carotid artery disease. The proportion of operated patients with symptomatic and asymptomatic ($\geq$ 70%) carotid artery disease were 85.9% (49/57) and 14.3% (6/42), respectively. One patient had coronary artery disease and was misdiagnosed. The diagnosis of carotid artery disease was therefore confirmed in 99% of the validation sample and symptomatic carotid artery disease in 57%

Validation of diagnosis of peripheral artery disease	One hundred of patients with diagnosis of peripheral artery disease were randomly selected for the validation procedure using patient record data. Among 100 patients, 69 had critical limb ischemia, 13 had acute limb ischemia, 15 had intermittent claudication, and one had asymptomatic peripheral artery disease. Of the 13 patients with acute limb ischemia, 12 had acute thrombotic occlusion and one had an embolic occlusion. Two patients had venous insufficiency and were thus misdiagnosed. The diagnosis of peripheral artery disease could therefore be confirmed in 98% of cases and symptomatic peripheral artery disease in 97%

Table S2. HR and 95% CI for incident atherosclerotic cardiovascular disease by adherence to diet quality index components.

Dietary components	Non-adherence	Adherence
**Saturated fat**	**>14E%**	**≤14E%**
Incident atherosclerotic disease	4130/19147	1728/7843
Age-and sex adjusted	1.00	1.00 (0.95-1.06)
Multivariable model	1.00	1.02 (0.96-1.09)
Mutually adjusted multivariable model[a]	1.00	1.06 (0.98-1.13)
**Polyunsaturated fat**	**<5 E% or >10 E%**	**5–10 E%**
Incident atherosclerotic disease	1767/8390	4091/18600
Age-and sex adjusted	1.00	1.00 (0.95-1.07)
Multivariable model	1.00	0.98 (0.93-1.04)
Mutually adjusted multivariable model[a]	1.00	0.99 (0.93-1.05)
**Sucrose**	**>10 E%**	**≤10 E%**
Incident atherosclerotic disease	1686/7566	4172/19424
Age-and sex adjusted	1.00	0.94 (0.88-0.99)
Multivariable model	1.00	0.95 (0.89-1.01)
Mutually adjusted multivariable model[a]	1.00	0.98 (0.92-1.05)
**Fiber**	**<2.4 g/MJ**	**≥2.4 g/MJ**
Incident atherosclerotic disease	4050/17944	1808/9046
Age-and sex adjusted	1.00	0.89 (0.84-0.94)
Multivariable model	1.00	0.95 (0.90-1.01)
Mutually adjusted multivariable model[a]	1.00	0.98 (0.91-1.06)
**Vegetables and fruit**	**<400 g/day**	**≥400 g/day**
Incident atherosclerotic disease	3793/16657	2065/10333
Age-and sex adjusted	1.00	0.89 (0.84-0.94)
Multivariable model	1.00	0.99 (0.93-1.05)
Mutually adjusted multivariable model[a]	1.00	1.06 (0.99-1.14)
**Fish**	**<300 g/week**	**≥300 g/week**
Incident atherosclerotic disease	3093/14726	2765/12264
Age- and sex adjusted	1.00	0.92 (0.87-0.97)
Multivariable model	1.00	0.97 (0.92-1.02)
Mutually adjusted multivariable model[a]	1.00	0.97 (0.92-1.02)

[a] Mutually adjusted multivariable model: Adjusted for age, sex, hypertension, diabetes mellitus, total energy intake, alcohol consumption, physical activity, smoking, education, body mass index, and mutual adjustment for the six diet quality index components.
